# Comprehensive Genome Analysis of *Neisseria meningitidis* from South America Reveals a Distinctive Pathogenicity-Related Prophage Repertoire

**DOI:** 10.3390/ijms232415731

**Published:** 2022-12-12

**Authors:** David Madariaga-Troncoso, Benjamin Leyton-Carcaman, Matias Garcia, Mikihiko Kawai, Michel Abanto Marin

**Affiliations:** 1 Scientific and Technological Bioresource Nucleus, Universidad de La Frontera, Temuco 4811230, Chile; 2 Laboratory of Molecular Applied Biology, Center of Excellence in Translational Medicine, Universidad de La Frontera, Temuco 4811230, Chile; 3 Department of Interdisciplinary Environment, Graduate School of Human and Environmental Studies, Kyoto University, Kyoto 606-8501, Japan

**Keywords:** IMSAR-11, pJS-B, filamentous phage, MDA, zot toxin, cc11 prophage, invasive meningococcal disease

## Abstract

*Neisseria meningitidis*, a bacterium that colonizes in the human nasopharynx, occasionally causes invasive meningococcal disease leading to meningitis or septicemia. Different serogroups and lineages (clonal complexes) are related to the occurrence and epidemiology of *N. meningitidis*. Despite vaccines for most serogroups, *N. meningitidis* lineages causing unusual clinical manifestations and a higher fatality rate compared to other lineages have been reported in South America. The present study focused on exploring the diversity of *N. meningitidis* prophages from South America and their relationship with the epidemiological variables of these strains. We found a high diversity of prophages among the different clonal complexes. By comparing them with previously described *N. meningitidis* phages and prophages, we revealed groups of prophages sharing similar compositions, which could be useful for prophage comparison in *N. meningitidis*. Furthermore, we observed a high correlation between the prophage content and epidemiological features, e.g., pathogenicity or clonal complex. Additionally, a distinctive filamentous prophage named here as IMSAR-11 (Invasive Meningococci from South America Related to cc11) was identified. Interestingly, two versions of IMSAR-11, circular and chromosomally integrated, were found. Overall, this study reinforces the importance of the genomic characterization of circulating *N. meningitidis* lineages to generate new targets for lineage monitoring, diagnosis, or appropriateness of vaccine development. Further studies are necessary to understand the role of these prophages in the persistence, dispersal, and virulence of *N. meningitidis* in the world.

## 1. Introduction

*Neisseria meningitidis* is extracellularly present in nearly 10% of the global population [1]. Generally, this interaction between bacteria and the host has a benign character. Still, when the commensal relationship ends, the presence of *N. meningitidis* can cause serious illnesses, among which the Invasive Meningococcal Disease (IMD) is one of the deadliest and leading causes of sepsis and meningitis worldwide [2].

In Chile, between 2000–2012, IMD incidences constantly decreased from 3.6 to 0.7 cases per 100.000 population. However, the fatality rate increased from 8% in 2009 to 27.0% in 2012 [3]. The increase in the fatality rate was mainly caused by the hypervirulent *N. meningitidis* serogroup W (MenW) belonging to the sequence type (ST)-11 clonal complex (cc11) [4]. From 2011–2019, the most frequent clonal complex of the W serogroup strains was cc11 (98.5% of the total) [5]. In other South American countries, the incidence of serogroup W has also been increasing, particularly in Argentina, Uruguay, and Brazil [6]. Although MenW cc11 is becoming dominant in South America, genomics studies focusing on its distinctive traits are scarce.

The genome of *N. meningitidis* is ~2.0–2.2 Mb in size and contains nearly 2000 genes [1,7]. Although the capsule is the primary virulence determinant of *N. meningitidis*, comparative genomics of various lineages have identified other genomic factors [8,9]. Neisserial genomes contain a repertoire of mobile genetic elements, including genomic islands, plasmids, and prophages, which allow increased genomic plasticity and could be associated with invasive lineages. For instance, it has been described that insertion sequences and prophages constitute up to 10% of the *N. meningitidis* genome [7].

Specific *N. meningitidis* prophages have been associated with the pathogenesis and invasiveness of the bacteria. These prophages have been described in lineages belonging to specific serogroups, clonal complexes, or geographic regions. For instance, the MDA (Meningococcal Disease Associated) prophage copies (Nf1-C1, Nf1-C2, Nf1-C3, and Nf1-C4), IHT-E, and phast-2 have been identified in the FAM18 strain belonging to serogroup C and cc11 [9,10,11,12]. The presence of MDA in the genome of *N. meningitidis*, via the production of viral particles, increases the colonization of *N. meningitidis* in the nasopharynx. Furthermore, MDAΦ infects nonpathogenic strains of *N. meningitidis* using the type IV Pili as a receptor through an interaction with the ORF6 adsorption protein. [13,14]. Other filamentous prophages, i.e., Nf2 and Nf3 [10] and Mu-like [15] prophages, have also been described in *N. meningitidis.* MDA/Nf1, Nf2, and Nf3 have a size of approximately 8 kb and possess a highly similar genomic organization, with proteins related to replication, structure, assembly, and a putative integrase gene assigned as a transposase [10]. IHT-E is a much larger prophage than the just mentioned, about 30 kb in size, and has proteins related to its replication, packaging, structure, and assembly [9].

The determining factors for the development of IMD are various and not thoroughly understood. This has caused that, notwithstanding the existence of vaccines, IMD outbreaks represent a serious global health concern [16]. Since prophages have been associated with advantageous phenotypes of host bacterial cells, studying them could allow us to better understand their relationship with *N. meningitidis* virulence [17]. Since the presence of prophages and their potential role in the epidemiology of circulating *N. meningitidis* lineages in South America have not been explored, this study aimed to contextualize invasive and epidemiological relationships of *N. meningitidis* with the prophage repertoire in *N. meningitidis* genomes from this geographic region.

## 2. Results

### 2.1. Phylogeny of N. meningitidis Isolates from South America

**Figure 1:**
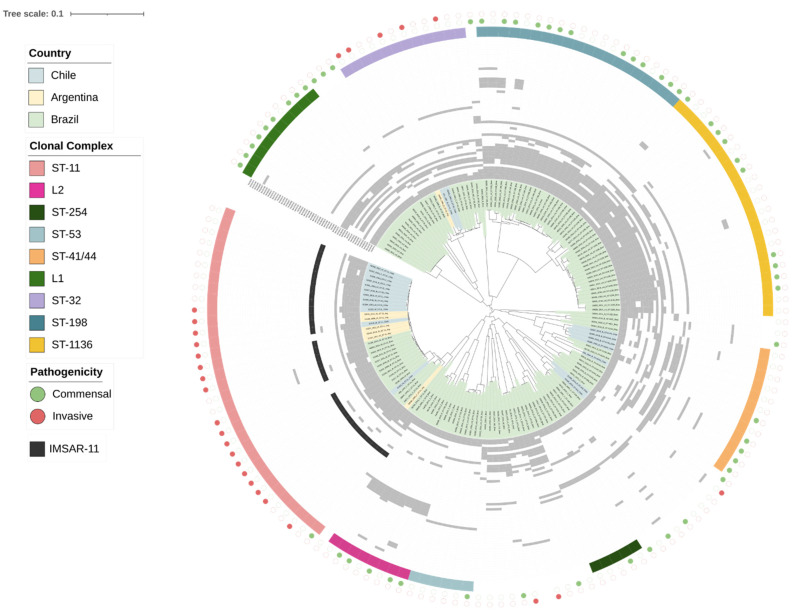
Prophage presence in *N. meningitidis* genomes from South America. The phylogenetic tree was constructed from 157 nucleotide genome sequences using the Harvest suite tool [18] and RAxML-NG [19]. Metadata were added on the phylogenic tree (from innermost to outermost: country, presence of the 56 prophage species—depicted in grey—, clonal complex, and pathogenicity). IMSAR-11 (Invasive Meningococci from South America Related to cc11) is depicted in dark grey. The grouping of prophages into families was carried out with the D6 formula of VICTOR [20]. A 0.4 LS-BSR score [21] was the threshold of a prophage presence. The order of prophage species from inner to outermost is the same as VICTOR’s output. When the pathogenicity of a genome is not described in the PubMLST database, the circle is not color-filled.

In order to establish the evolutionary relationship of *N. meningitidis* from South America, we reconstructed a phylogenetic tree comprising 157 genomes isolated from 1973 to 2016 in Chile, Argentina, and Brazil (Figure 1, Table S1). Eight clades corresponding to different clonal complexes were identified. No clear phylogenetic segregation was observed between the carrier (commensal) and invasive strains. In each country, more than one serogroup and clonal complex were observed, and we found regular correspondence between the clonal complexes and phylogenetic clades. The cc11 includes isolates from Chile, Brazil, and Argentina. The genomes linked to invasiveness are mainly represented by cc11, followed by cc32, cc41/44, and cc5. On the other hand, clonal complexes linked to commensal genomes were more diverse and represented mainly by cc1136, cc254, cc53, and cc198 (Figures S1 and S2). We found two clades that were not associated with any clonal complex and were mainly associated with commensal genomes. We designated those clades as clonal complex L1 and L2 (Lineage 1 and Lineage 2). ccL1 comprised genomes assigned as capsule null locus (cnl) meningococci, which are more closely related to serogroup B (cc32, cc35, cc269). ccL2 contained genomes corresponding to the Z serogroup, which is more closely associated with serogroup C of cc11 and serogroup W of cc11 (Figure 1).

### 2.2. High Viral Variability in N. meningitidis

**Figure 2:**
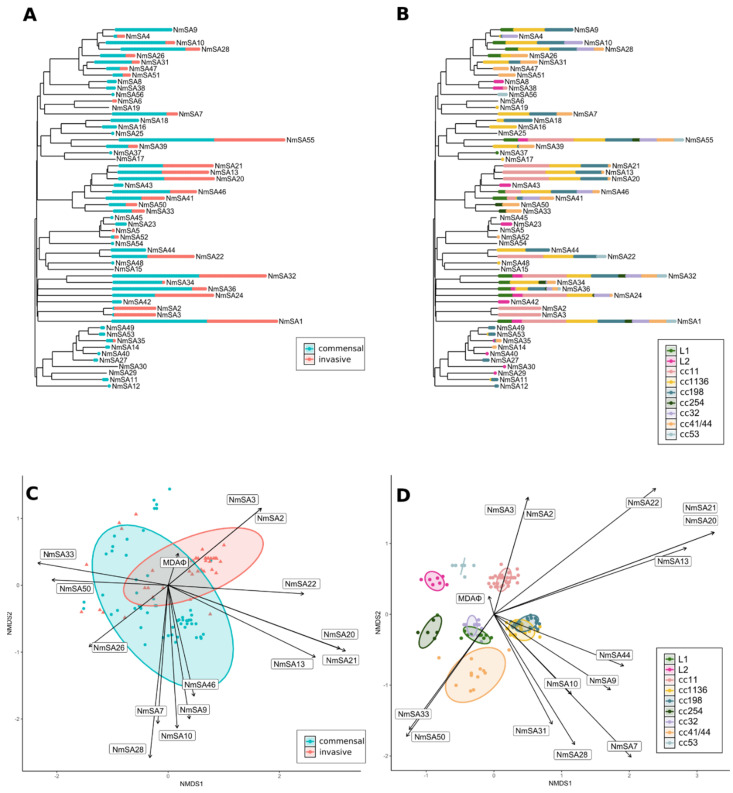
Prophage content related to epidemiological features in *N. meningitidis* from South America. The 56 species identified by the D6 VICTOR formula were used for this analysis [20]. The phylogenetic tree was generated by D6-VICTOR and rooted at the midpoint. Prophage abundance in the genomes of *N*. *meningitidis* is represented according to pathogenicity (**A**) and clonal complexes (**B**). Non-parametric multidimensional scaling (NMDS) analyses, based on prophage content, show the relationship between prophage structure and pathogenicity (**C**) and clonal complexes (**D**). In the NMDS plots, only significant prophages (vectors) with an R^2^ greater than 0.4 are represented, except for MDA, which was not significant and was only plotted as an initial reference point. Vectors are proportional to R^2^.

Initially, 430 viral sequences were identified in the 157 bacterial genomes, with an average of ~three prophages per *N. meningitidis* genome. Subsequently, based on their nucleotide similarity, the total number was reduced to 62 representative prophages. We inferred phylogenies from these 62 representative prophage genomes, obtaining estimates for taxon boundaries at distinct ranks using VICTOR (Figure 2A,B). Based on amino-acid sequences analysis, the OPSIL D6 clustering formula identified 56 potential species, 14 genera, and three families of viruses, also generating the tree with the highest average branch support among the three distance formulas used by it [20]. Considering that some of the prophages found are part of contigs derived from short-read sequencing technologies, only predicted prophages > 4 kb were included in our analyses.

Of the 56 viral species, 20 corresponded to lysogenic (integrated proviruses), 35 were assigned as lytic, and one was not categorized (Table S2). It is important to note that VIBRANT [22] classifies a prophage as lysogenic if it is detected as an integrated viral sequence or if an associated integrase is present. Additionally, it predicts as lytic all prophages that are not considered lysogenic. The prediction and classification of lysogenic prophages performed by VIBRANT should be accurate. However, lytic prophages could also be lysogenic prophages lacking some of the features detailed above. Therefore, we consider that the prophages assigned as lytic in this study should be viewed with caution. Future functional characterizations will be necessary to reveal the type of prophage they represent. The prophages were named using as prefix *NmSA* (denoting a virus of *Neisseria meningitidis* from South America). The suffix number was assigned arbitrarily by the number of the species provided by the D6 formula of VICTOR (Table S1).

### 2.3. Correlation of Prophage Repertoire with Clonal-Complexes and Pathogenicity of N. meningitidis

We found a relationship between the presence of prophages and pathogenesis (PERMANOVA test; *p*-value < 0.0001) and between prophages and clonal complex (*p*-value < 0.0001) (Figure 1 and Figure 2C,D). Furthermore, there were significant differences between the prophage content and genogroup (*p*-value < 0.0001) and country (*p*-value < 0.0001).

Further analyses revealed the importance of specific prophages with clonal complexes and pathogenicity. It was found that prophage diversity is more significant in commensal strains than in invasive ones and that prophage regions are widely distributed in both commensal and invasive genomes (Figure 2C and Figure S1). Prophages that correspond to the NmSA9 clade (NmSA9, NmSA10, and NmSA28) were more associated with commensal strains (Figure 2C) and cc1136 (Figure 2D). NmSA2 and NmSA3 are related mainly to invasiveness and cc11 strains (Figure 2B,D). NmSA13, NmSA20, and NmSA21 were indistinctly distributed among commensal and invasive strains. On the other hand, except for NmSA23, NmSA43, NmSA8, and NmSA56, all prophages were distributed in at least two clonal complexes (Figure 2D). ccL1 and ccL2 (Figure 1) were assigned according to the phylogeny obtained from the *N. meningitidis* genomes. Interestingly, NmSA8 and NmSA23 were found exclusively in the ccL2 clade (Figure 2B).

Random forest models were used to determine the most important epidemiological variable related to the prophage content of the genomes. Interestingly, the models were 100% assertive with the epidemiological-feature classification (kappa test = 1), emphasizing the relationship between prophage structure and pathogenicity and prophage patterns among different clonal complexes. According to the average Gini index, these prophages are more related to the clonal complex rather than the invasiveness of *N. meningitidis*, given that it ranks NmSA2 and NmSA3, second and third, respectively. Meanwhile, regarding pathogenicity, it ranks NmSA2 and NmSA3, as third and fifth, respectively (Figure S3).

### 2.4. Prophage Groups Related to Pathogenicity (Commensal or Invasive) and Clonal Complexes Are Similar in Structure and Function

**Figure 3:**
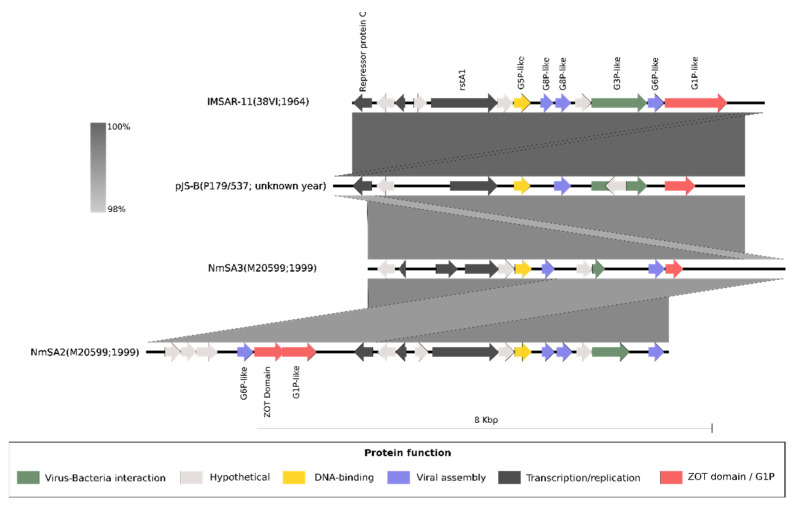
Prophage genome comparison among IMSAR-11 members. The strain name of the bacterium host and the isolation year are indicated in parentheses. Colors for genes are based on the analysis of HHpred [23]. The shaded lines (in grey) depicts nucleotide similarities between the sequences. NmSA2 and NmSA3 were found in the same genome of M20599 (GCA_001407225.1; PubMLST id: 31322; Chile). The reverse complement of pJS-B (NC_004758.1; Norway) and NmSA2 are depicted. IMSAR-11 of 38VI (BioSample: SAMEA678244; PubMLST id: 349; the United States), pJS-B, and NmSA3 are circular forms, whereas NmSA2 is flanked by chromosomal regions in the *N. meningitidis* genome.

Besides the phylogenetic relationship of specific prophages, a strong correlation between some of them and pathogenicity/lineages was noted (Figure 2C,D). The prophage structures, based on their similarities, were explored and revealed that: (i) NmSA2 and NmSA3 were associated with invasive cc11 genomes; (ii) NmSA13, NmSA20, and NmSA21 were related to invasive and commensal genomes from cc11, cc1136, cc198, and cc41/44; (iii) NmSA9, NmSA10, and NmSA28 were related to commensal genomes from cc254, cc1136, cc198, and cc32. The groups mentioned above showed similar synteny (Figure 3, Figures S4 and S5). NmSA7 and NmSA46 presented discrepant sizes and structures compared to NmSA9, 10, and 28.

### 2.5. The Majority of Prophages Predicted Are Related to Prophages Previously Described in the Literature

**Figure 4:**
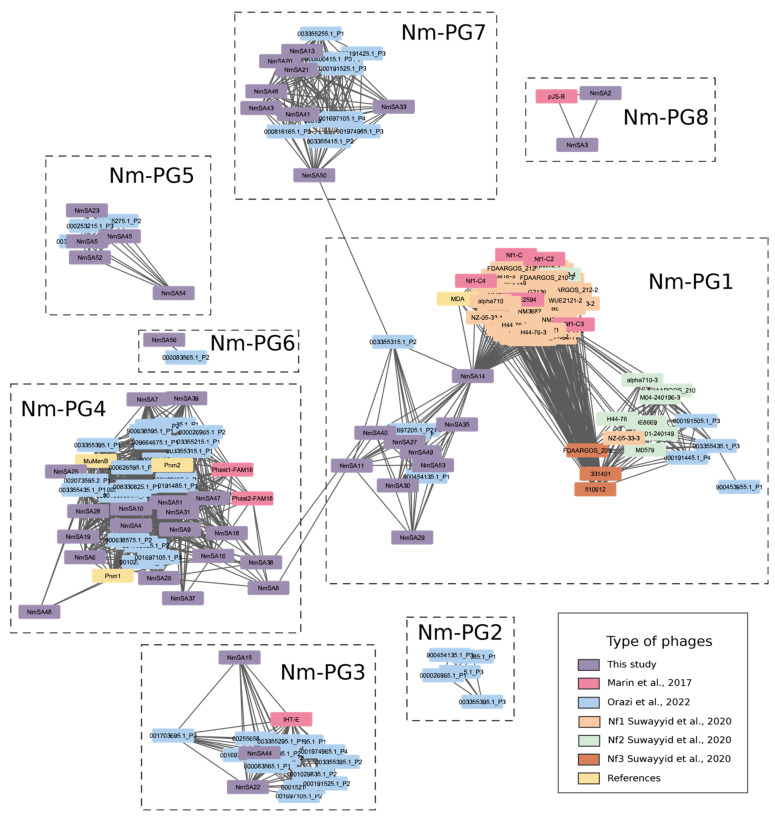
Comparison of the predicted prophages with prophage sequences found in the literature. The protein network was constructed using vConTACT2 [24] and visualized with Cytoscape [27]. The Nm-PG (*N. meningitidis*-Prophage Group) groups of prophages were designated according to their protein content. Each prophage is labeled in color according to references of comprehensive studies. The prophages of Marin et al. (2017) [12] include strains of *N. meningitidis* from Brazil. The prophages predicted by Orazi et al. (2022) [25] represent a large-scale bioinformatics approach to prophage diversity in *Neisseria spp*. On the other hand, we also included prophages studied by Al Suwayyid et al. (2020) [28], who investigated filamentous prophages classified as MDA/Nf1, Nf2, and Nf3, according to the nomenclature proposed by Kawai et al. (2005) [10]. Finally, we included other known prophages, i.e., PS_1 reported by Bettencourt et al. (2022) [29], MDA reported by Bille et al. (2005) [11], and Mu-like prophages, such as Pnm1, Pnm2, and MuMenB reported by Masignani et al. (2001) [15]. More data related to this figure are available at GitHub (https://github.com/Leytoncito/ProphagesNM/blob/main/README.md).

The protein network analysis revealed that most of the prophages predicted in this study were related to prophages found in the literature (Figure 4). The protein network was structured into 16 viral clusters, which are roughly equivalent to ICTV genera [24]. 51/56 prophages predicted by us were distributed in 12 viral clusters. Based on a visual inspection, we arbitrarily regrouped the 16 viral clusters into nine groups (*Neisseria meningitidis*-Prophage Group, or Nm-PG).

Nm-PG1 includes the filamentous prophages MDA/Nf1, Nf2, Nf3 [10,11], and nine prophages predicted in this study (Figure 4). Nm-PG2 includes exclusively prophages predicted by Orazi et al. (2022) [25]. Nm-PG3 has NmSA22, NmSA44, NmSA15 (~40 kb), prophages predicted by Orazi et al. (2022) [25], and IHT-E [9]. Nm-PG4 contains most of the prophages predicted in this study (*n* = 21), which would be associated with MuMenB, including Pnm1, Pnm2, Phast1-FAM18, and Phast2-FAM18 mu-like prophages [12,15]. Nm-PG5 includes prophages ~41 kb in size and predicted by Orazi et al. (2022) [25] and by us. Nm-PG6 is formed by NmSA56 (~23 kb) and 000083565.1_P2 (~26 kb). Nm-PG7 is formed by NmSA13, NmSA20, and NmSA21, which are highly distributed in both commensal and invasive *N. meningitidis* strains. Nm-PG8 includes NmSA2 and NmSA3 (both described here as versions of IMSAR-11); the latter group also includes pJS-B [26].

### 2.6. Genomics of IMSAR-11

NmSA2 and NmSA3 were identified in the same genomes. These two syntenic prophages have highly similar nucleotide and amino acid content (Figure 3). The NmSA2 version was identified as an integrated prophage; meanwhile, NmSA3 was in a circular form. Considering this, we named both versions (NmSA2 and NmSA3) as prophage IMSAR-11 (**I**nvasive **M**eningococci from **S**outh **A**merica **R**elated to cc**11**). Through a visual exploration of complete genomes (See Section 4.1), we noticed that the chromosomally integrated version of IMSAR-11 is flanked by the same genomic context and in some genomes present as a tandem-duplicated version (Figure S6). In the case of MDAΦ, the copy number is related to its pathogenic potential [30], and in the hyperinvasive lineages, the prophage has more than one copy [14]. It is unclear why IMSAR-11 would contain copies or versions of the prophage, but it might relate to its replication or dissemination, as occurs with MDAΦ. To estimate IMSAR-11 activity, we applied a method of the prophage:host read coverage ratio using PropagAtE [31] with a genome (GenBank: CP017257), which contains a single non-circular chromosomal copy of the prophage. The read coverage of IMSAR-11 was five-times more than the baseline read coverage of the host, suggesting IMSAR-11 is an actively replicating prophage.

IMSAR-11 is predicted to have 11–13 *orf*s (Figure 3) and has a genetic structure similar to that seen in filamentous prophages such as MDA/Nf1. However, there was no nucleotide and amino-acid similarity between IMSAR-11 and other known filamentous prophages in *N. meningitidis*. The protein network (Figure 4) showed that IMSAR-11 is part of the Nm-PG8 prophage cluster, distinct from MDAΦ and Nf prophages (Nm-PG1). However, five proteins share high structural homology with ORF2, ORF4, ORF5, ORF7, and ORF8 of MDAΦ from strain Z2491 (Figure S7). The protein analysis of IMSAR-11 showed viral structure-related proteins, e.g., capsid and tegument proteins, and replication/transcription proteins (Figure 3 and Table S3). G1P of IMSAR-11 also contains a *zot*-like domain. Moreover, comparative structural analysis between G1P (from 38VI; PubMLST ID 349) and ORF8 protein from MDAΦ (from isolate Z2491) showed a similar homology (Figure S7). An attachment protein G3P, similar to the one in bacteriophage M13, was also found. In M13, this protein performs an essential role both in the penetration of the viral genome into the bacterial host and in the extrusion process (UniProt id: P69168).

### 2.7. Global Distribution and Epidemiology of IMSAR-11

IMSAR-11 was found in 4110/27116 *N. meningitidis* genomes in the PubMLST database (Table S4, Figure S8). 4002/4110 genomes (98.84%) that presented IMSAR-11 belonged to cc11, followed by cc8 with a 0.99% (40/4110) of the cases, then by cc32 and cc41/44 with 0.05% (2/4110), and lastly by cc22, cc23, and cc1157 with 0.02% each one (1/4110). Of the total cc11 genomes (*n* = 6276), IMSAR-11 was prevalent in South America, North America, Europe, Asia, Africa, and Oceania, respectively, in the 88.38%; 86.82%; 65.70%; 42.42%; 21.79%; and 87.36% of the total of cc11 genomes per continent. The distribution among serogroups revealed that 62.25% of the genomes containing IMSAR-11 were part of the W serogroup, followed by 34.45% of the C serogroup, 2.95% of the B serogroup, and 0.35% part of the Y serogroup. IMSAR-11 prevalence in invasive genomes from South America was around 99.44%. Similar results were observed in Oceania, with 93.42%, Africa (81.86%), and Europe (77.11%). These percentages were lower in Asia and North America (41.86%) and North America (50.65%).

### 2.8. MDA Presence in South America

Considering MDAΦ role in the increase of invasiveness [13,32], we examined its presence in all original 157 *N. meningitidis* genomes and found that it was contained in seven genomes. When considering 442 genomes from the PubMLST database, its presence increased up to 59 genomes (Table S4). MDA/Nf1 presence in South America dates back to 1976 (PubMLST ID: 39837), from a Men C cc11 isolate from Brazil. The latter is interesting because IMSAR-11 presence in South America dates back to 1973 (PubMLST ID: 34571) from Brazil, also in a MenC cc11 isolate (note that 38VI was isolated in 1964 but from the United States). This suggests a co-existence of both prophages in South America for at least four decades.

## 3. Discussion

This is the first systematic study that explores the prophage content of *N. meningitidis* isolates from South America. The analysis revealed a relationship between the prophage content and clonal complexes, i.e., prophage content varies depending on the clonal complex. Furthermore, specific prophages are more related to invasive strains rather than commensal ones, and vice versa. Various analyses were performed to deepen the relationship between prophages, pathogenicity (invasive/commensal), and the different clonal complexes, which will be discussed below.

### 3.1. Known and Novel Predicted Prophages of N. meningitidis from South America

Other studies have recently delved into the prophages of *N. meningitidis* [25,29]. Bettencourt et al. (2022) [29] associated their cc11 isolates, which included a variety of serogroups, with the PS_1 prophage of *Pseudomonas*. In our study, the PS_1-like prophage resulted in a singleton occurrence; namely, it was unrelated to the prophages in the protein network. Our study was based on the relation of the viral elements at a protein level, while Bettencourt et al. (2022) [29] identified the PS_1-like presence based on the PHASTER Tool [33], which performs analyses based on DNA sequences. Considering that in *N. meningitidis* phase variation occurs [34,35], the non-detection of PS_1 might be due to our different approach. Further studies are necessary to elucidate the presence and function of this genetic element in *N. meningitidis* isolates from South America. On the other hand, Orazi et al. (2022) [25] predicted, in a bioinformatic approach, a high number of prophages in *N. meningitidis*. The prophages predicted by Orazi and collaborators [25] were noticeably related to the predicted ones in this study (Figure 4).

Most of the predicted prophages were Mu-like prophages. Mu-like prophages of *N. meningitidis* possess mosaic structures with intercalated segments not related to the DNA prophage, which in turn encode surface-exposed antigens [15]. Nm-PG4, which includes the Mu-like, was the most variable group. Gene diversity of Mu-like prophages, in terms of protein content, could be explained because those regions would be acting as gene exchange platforms or recombination matrices and would not represent a type of parasitic DNA in the sense of not having a relevant role in the biology of *N. meningitidis*, as mentioned in a previous study [36]. Other prophage groups with similar genome length (~40 kb), such as Nm-PG3 (associated with IHT-E) and Nm-PG5, were more similar in terms of proteins and genera, which suggests they would be more structurally conserved than Mu-like prophages, and possibly be acting as a gene reservoir. Even though IHT-E has been associated with hyper-invasive strains [36], our results indicate that there is no relationship between prophages associated with IHT-E (Nm-PG3) and the pathogenicity of *N. meningitidis* in South America.

Previous studies have associated MDA/Nf1 and Nf2 prophages with an advantage for the invasiveness of *N. meningitidis* [11,13,14]. However, MDA/Nf1, Nf2, and Nf3 prophages did not significantly correlate with invasiveness in South America. Interestingly, within Nm-PG1, NmSA14 shares proteins with the Nf viral cluster, which includes MDA. NmSA14 is not part of the same viral cluster as the MDA/Nf1; however, a detailed inspection showed the MDA/Nf1 structure was contained in the larger prophage NmSA14, which suggests the presence of mosaic structures and probably the existence of these prophages being infected by other satellite prophages. This has been previously seen in different bacteria, such as *Vibrio cholerae*, where the CTX prophage harbors RS1 and TLC prophages [37].

Nm-PG7 was an evenly distributed group containing both invasive and commensal *N. meningitidis* genomes. Interestingly, they constituted a single prophage cluster and a single VICTOR genus. As with other lineage-specific prophages, the prophage maintenance in this lineage could be associated with restriction-modification systems modulating homologous recombination [38] and could represent a stable gene pool reservoir in *N. meningitidis*.

IMSAR-11 is highly similar to pJS-B (Figure 3). Nm-PG8 contained IMSAR-11 clustered with pJS-B; the latter was described as a plasmid more than two decades ago [26]. Interestingly, similar to us, Claus et al. detected two versions of the element; one was inserted into the chromosome; meanwhile, the other was present in a circular form. Furthermore, the authors also detected its correlation with cc11 (ET-37) and identified a conserved specific region IR1/IR2 that, according to them, might serve as the chromosomal integration site for pJS-B. Although pJS-B was initially described as a plasmid, the similarities found with IMSAR-11 in this study demonstrate that pJS-B is a circular form of a prophage belonging to the IMSAR-11 group.

Although MDA/Nf1 and IMSAR-11 belong to different prophage groups, comparative analyses showed that IMSAR-11 might be a filamentous prophage with a similar structure and functionality. Indeed, we found similarities at the protein structural level between G1P of IMSAR-11 and ZOT (ORF8) of MDAΦ (Figure S7). Our comparative results showed a more significant conservation of amino-terminal cytoplasmic domains, which could suggest a role in viral assembly. IMSAR-11 and MDAΦ also shared structural similarities with another four proteins (Figure S7): G6P-like (ORF7), G8Pb-like (ORF5), G8Pa-like (ORF4), and G5P-like (ORF2). Moreover, compared with MDA/Nf1, another four hypothetical proteins were found exclusive for IMSAR-11. Considering the non-detectable identity at the nucleotide level and the low similarity at the amino acid level, our results suggest that these elements could derive from a distant common ancestor but could also be the product of convergent evolution. Further extensive comparative studies will be necessary to unravel these elements’ origin and functional persistence.

G3P plays an important role in the initial interaction between the prophage and cell host (UniProt id: P69168). G3P from 38VI is similar to the C- terminal domain of TspB, a known virulence factor of *Neisseria meningitidis* [39]. Müller and collaborators found that by knocking out G3P genes (ORF6 of MDA), *N. meningitidis* did not form large aggregates in biofilm and that TspB mediates biofilm formation in human serum [40]. Further analyses are necessary to elucidate if IMSAR-11 G3P performs a similar function. Different versions of IMSAR-11 that possess truncated or non-coding versions of the G3P protein could be related to the regulatory processes of the prophage or its interaction with the host. Moreover, since this region of the prophage genome is rich in homopolymers, it could promote mechanisms to alter the expression of surface-exposed proteins through phase and antigenic variation [41]. On the other hand, the variations observed could have arisen through the PacBio assembly process of genomes, such as M20599. However, the similar IMSAR-11 structure is found in other genomes sequenced by Illumina and PacBio such as CP017527 (USA), CP045961 (Australia), CP009422 (Chile), and CP009418 (Brazil).

### 3.2. Epidemiology of IMSAR-11

The blast search of IMSAR-11 revealed its high conservation, integrity, and global distribution (51 countries from all continents) among *N. meningitidis* genomes. The presence of this prophage dates back to 1964, from a MenB cc11 isolate (38VI) of the United States (PubMLST ID: 349). The latter might account for the importance of this prophage in the emergent hyperinvasive *N. meningitidis* cc11 at a global evolutionary scale for approximately six decades. Furthermore, the fact that IMSAR-11 is almost exclusively present in cc11 likely could be related to a host-specific receptor or to a resistance mechanism against other prophages different from those of cc11.

Our results suggest that IMSAR-11 would be an active prophage, which could account for an important role in the biology of the *N. meningitidis* from cc11. However, these findings were found by only analyzing one representative genome. Therefore, it is necessary to analyze more genomes to evaluate the veracity and importance of this finding.

In South America, all cc11 invasive strains, except one, harbored IMSAR-11. When the presence of the prophage was explored in the rest of the world (North America, Africa, Europa, Oceania, and Asia), the results were similar, except for North America and Asia. The lower percentage of invasive isolates in these two continents is possibly related to genomes missing information related to their invasive or commensal source. Even though IMSAR-11 is predominantly associated with invasive *N. meningitidis* strains, it is also present in commensal isolates. The latter might be attributable to the fact that the invasive state depends on many factors, such as the state of the host’s immune system, its microbiome, and the virulence capacity of *N. meningitidis* [42,43,44,45]. In this study, we identified a distinctive repertoire of prophage related to circulating lineages in South America. The low IMSAR-11 prevalence in the African and Asian cc11 isolates might also be related to lineages more prevalent than cc11 in those regions. Similarly to IMSAR-11, the prevalence of other invasive lineages of *N. meningitidis* would also be related to the prominence of certain prophages, as occurs with MDA in Africa [32].

### 3.3. Impact, Limitations, and Future Perspectives

The relevance of this study lies in the fact that, for the first time in South America, the prophage content of *N. meningitidis* was systematically explored. Using different genomic, epidemiological, and integrative approaches, we were able to characterize prophages related to specific variables, including the invasive and commensal state of *N. meningitidis*. Moreover, we found that the prophage content of *N. meningitidis* is going to differ depending on the clonal complex, serogroup, and country.

We suggest that the predicted prophages could be used as epidemiological markers. The random-forest analysis revealed that IMSAR-11 might serve as a cc11 predictor. This idea was reinforced by the blastn search of IMSAR-11, which showed its high conservation in invasive cc11 genomes worldwide. On the other hand, NmSA9 and NmSA10 could also be used as commensal markers and, along with IMSAR-11, could differentiate commensal from invasive strains and explain, in part, both types of pathogenicity.

An utterly satisfactory tool for prophage prediction does not exist. We chose VIBRANT, a software that has shown a lower rate of false positives identification and a greater ability to maximize the recovery of viromes [22]. Considering that each fragment of a prophage DNA can be affected by mosaicism and that at the protein level, older ancestry relationships can be obtained [46,47], this study was carried out mainly with a protein analysis approach. Prophage prediction is going to differ depending on the analysis approach that is taken, i.e., if the study is focused on prophage proteins or nucleotides. We predicted previously identified prophages described in the literature, except for PS_1 and MDAΦ [14,29]. This might be because VIBRANT includes protein annotations in its prediction algorithms, so a change in the reading frame of DNA would lead to inconsistencies with the amino acid sequences. Considering that these frameshifts have already been described [34,35], they might be affecting the reading of the DNA of, for instance, MDA. The taxonomic classification of prophages is difficult due to mosaicism. We tried to get a closer look into the taxonomy of the predicted prophages by performing a protein network analysis using vConTACT2 and the INPHARED database [48], but we could not classify most of them (Table S2). Therefore, further taxonomic studies are needed.

## 4. Materials and Methods

### 4.1. Genomes Recovery

Initially, a total of 157 *N. meningitidis* genomes from Chile (*n* = 21), Argentina (*n* = 7), and Brazil (*n* = 129) were recovered from the public database PubMLST (https://pubmlst.org, accessed on 30 September 2020). Subsequently, to measure the scope of our results, we explored the rest of the genomes of South America and the world deposited in PubMLST (*n* = 27,116, by 1 June 2022. By this date the number of genomes of South America increased up to 442). We used M20599 (PubMLST ID 31322; Chile) and 38VI (PubMLST 349; the United States) isolates throughout this manuscript as representative genomes of South America. In addition, we included the complete genomes from GenBank (CP017257, CP009418, CP045961, and CP009422) to carry out prophage characterization and a reinforcement of our findings. Metadata related to the genomes is described in Table S1.

### 4.2. Bioinformatics Analysis

After the core-genome alignment obtained by the Harvest suite tool [18], RaxML-NG was used to infer the evolutionary relationship of the genomes [19]. RaxML-NG analysis was carried out with 100 replicates of bootstrap using the GTR + G + FO model. VIBRANT [22] was used for detecting, annotating, and characterizing prophages. To decrease prophage redundancy, CD-HIT [49] was used with a cutoff of 75% clustering threshold. A phylogenetic tree of the representative prophage sequences was generated with Virus Classification and Tree Building Online Resource (VICTOR) [20]. Finally, the prophage content was studied using LS-BSR [21] and iTOL [50]. Only predicted prophages > 4 kb were considered for comparative analyses.

### 4.3. Data Analysis and Visualization

The analysis and data visualization were performed using the R packages ggplot2 (https://github.com/tidyverse/ggplot2), vegan (https://github.com/vegandevs/vegan), randomForest (https://cran.r-project.org/web/packages/randomForest/index.html), and phyloseq (https://joey711.github.io/phyloseq). A phylogenomic exploration was carried out with iTOL [50], while the schematic representations of the structures of the prophages were done using Easyfig [51].

The Initial LS-BSR matrix was transformed into a binary matrix considering the presence of prophages with a LS-BSR score equal to or superior to 0.4. A Jaccard distance matrix was calculated to evaluate the relationship between the prophage content and the epidemiological characteristics of *N. meningitidis*, and a permutational multivariate ANOVA (PERMANOVA) test was applied. Subsequently, to visualize the relationship between the absence/presence of the prophages and epidemiological variables, a non-parametric dimension reduction was applied utilizing NMDS. We delimited a *p*-value < 0.05 and R^2^ > 0.4 to graphically represent the most significant prophages and most correlated epidemiological variables. Machine learning models were generated to determine the most important epidemiological variable related to the prophage content. For this study, the Random-Forest algorithm (with 1000 decision trees) was used [52], and the model efficiency was evaluated using the kappa concordance test. Furthermore, a phyloseq object, based on the D6 formula from VICTOR and prophage content, was generated using the R phyloseq library.

### 4.4. Network Analysis

To contextualize our predicted prophages, we compared them with previously described ones using vConTACT2 [24]. The reference phages and prophage of *N. meningitidis* included in this study are MDAΦ (present in several serogroups), pnm1, pnm2 of the Z2491 (serogroup A, CGA_000009105.1) strain, MuMenB prophage of the MC58 (serogroup B, GCA_000008805.1), IHT-E, pJS-B, the Nf prophage family, prophages predicted by Orazi et al. (2022), and the PS_1 prophage [9,10,11,12,25,26,28,29]. Furthermore, to infer a taxonomy, the predicted prophages were compared using the INPHARED (Infrastructure for a Prophage Reference) database [48]. The protein network was visually inspected and ordered with Cytoscape [27] using the edge-weighted spring-embedded layout algorithm.

### 4.5. Bioinformatics Analysis of IMSAR-11

The results revealed an invasiveness-related prophage, which we named IMSAR-11 (**I**nvasive **M**eningococci from **S**outh **A**merica **R**elated to cc**11**. See Results and Discussion). To elucidate the presence of IMSAR-11 in more recent genomes and to get a broader scope of its role in the pathogenicity of *N. meningitidis*, a blastn search encompassing all *N. meningitidis* genomes in the world (27,116 genomes in total) was carried out using the BLAST plugin of the PubMLST database (1 June 2022). The presence of IMSAR-11 in a genome was defined with a cutoff of above 90% coverage and 98% similarity. Furthermore, IMSAR-11 presence was contrasted concerning clonal complexes, the distribution within cc11 genomes, and serogroups.

The structural prediction of IMSAR-11 proteins was made using HHpred [23]. Models of their structures were predicted and generated using AlphaFold [53,54] and visualized with PyMOL (https://pymol.org/2/). PropagAtE [31] was used to estimate the prophage activity of IMSAR-11 by the prophage:host read coverage ratio and corresponding effect size. We used UGENE [55] to visually explore the genome context of IMSAR-11.

## 5. Conclusions

Our findings suggest that prophage content and distribution are related to the epidemiology of *N. meningitidis* and that the distinctive prophage repertoire is a consequence of the evolution of the meningococci lineages. Since cc11 has been acquiring epidemiologic relevance in the last decades, the genomic understanding of *N. meningitidis* belonging to cc11 is crucial. Moreover, IMSAR-11 could play an important role in the invasiveness of cc11 around the world, especially in South and North America, but in other continents as well. On the other hand, we named eight groups of characteristic prophages in *N. meningitidis*, which are made up of prophages sharing similar protein content. Members of Nm-PG7 would be widely distributed in strains of *N. meningitidis*. In addition, members of Nm-PG1 and Nm-PG8 would be associated with invasive lineages, and members of Nm-PG4 would be related to commensal strains.

## Supplementary Materials

The following supporting information can be downloaded at: https://www.mdpi.com/article/10.3390/ijms232415731/s1. A repository is available on GitHub (https://github.com/Leytoncito/ProphagesNM, accessed on 14 October 2022) with all the bioinformatics and statistical analysis for the total reproduction of this study. In this Github, we also make available the prophage sequences predicted by this study in different formats.
