# Supplementary material for: Comprehensive Genome Analysis of Neisseria meningitidis from South America Reveals a Distinctive Pathogenicity-Related Prophage Repertoire
Source: Int J Mol Sci. 2022 Dec 12;23(24):15731. doi: 10.3390/ijms232415731 (PMC9779448; doi:10.3390/ijms232415731)
Supplement: Supplementary file 1 [file ijms-23-15731-s001.zip › ijms-1999247-supplementary.pdf]

Supplementary Section: Comprehensive genome analysis of *Neisseria meningitidis* from South America reveals a distinctive pathogenicity-related prophages repertoire

David Madariaga-Troncoso[1], Benjamín Leyton-Carcaman[1], Matías García[2], Mikihiko Kawai[3], Michel Abanto-Marín[1]*

[1]*Scientific and Technological Bioresource Nucleus, Universidad de La Frontera, Temuco, Chile*

[2] *Laboratory of Molecular Applied Biology, Center of Excellence in Translational Medicine, Universidad de La Frontera, Temuco, Chile.*

[3] *Department of Interdisciplinary Environment, Graduate School of Human and Environmental Studies. Kyoto University, Kyoto, Japan.*

Correspondence: *mfabanto@gmail.com*.

Contents

Supplementary Data	Page
Table S1	1
Table S2	5
Table S3	7
Tables S4	8
Figure S1	10
Figure S2	10
Figure S3	11
Figure S4	11
Figure S5	12
Figure S6	12
Figure S7	13

Table S1. Details of studied genomes

PubMLST id	isolate	country	year	disease	species	capsule group	ST	clonal complex
441	8680	Chile	1987	invasive (unspecified/other)	Neisseria meningitidis	B	32	ST-32 complex
468	BRAZ10	Brazil	1976		Neisseria meningitidis	C	11	ST-11 complex
84	IAL2229	Brazil	1976		Neisseria meningitidis	A	5	ST-5 complex
650	M40/94	Chile	1994	invasive (unspecified/other)	Neisseria meningitidis	B	41	ST-41/44 complex
26029	8733	Chile	1980		Neisseria meningitidis	B	32	ST-32 complex
26034	27/86	Brazil	1986		Neisseria meningitidis	B	33	ST-32 complex
26035	270/94	Argentina	1994		Neisseria meningitidis	B	1880	ST-32 complex
26037	71/87	Brazil	1987		Neisseria meningitidis	B	33	ST-32 complex
26038	84/89	Brazil	1989		Neisseria meningitidis	B	32	ST-32 complex
31148	M14 240411	Argentina	2008	invasive (unspecified/other)	Neisseria meningitidis	W	11	ST-11 complex
31149	M14 240412	Argentina	2010	invasive (unspecified/other)	Neisseria meningitidis	W	11	ST-11 complex
31150	M14 240413	Argentina	2011	invasive (unspecified/other)	Neisseria meningitidis	W	10856	ST-11 complex
31151	M14 240414	Argentina	2011	invasive (unspecified/other)	Neisseria meningitidis	W	10857	ST-11 complex
31152	M14 240415	Argentina	2012	invasive (unspecified/other)	Neisseria meningitidis	W	11	ST-11 complex
31155	M14 240416	Brazil	2011	invasive (unspecified/other)	Neisseria meningitidis	W	11	ST-11 complex
31156	M14 240417	Brazil	2011	invasive (unspecified/other)	Neisseria meningitidis	W	11	ST-11 complex
31157	M14 240418	Brazil	2011	invasive (unspecified/other)	Neisseria meningitidis	W	11	ST-11 complex
31158	M14 240419	Brazil	2010	invasive (unspecified/other)	Neisseria meningitidis	W	11	ST-11 complex
31159	M14 240420	Brazil	2008	invasive (unspecified/other)	Neisseria meningitidis	W	11	ST-11 complex
31160	M14 240421	Brazil	2009	invasive (unspecified/other)	Neisseria meningitidis	W		
31161	M14 240422	Brazil	2008	invasive (unspecified/other)	Neisseria meningitidis	W	11	ST-11 complex
31317	NM3686	Brazil		invasive (unspecified/other)	Neisseria meningitidis	W	11	ST-11 complex
31319	M10208	Chile		invasive (unspecified/other)	Neisseria meningitidis	W	11	ST-11 complex
31320	NM3687	Brazil		invasive (unspecified/other)	Neisseria meningitidis	W	11	ST-11 complex
31322	M20599	Chile		invasive (unspecified/other)	Neisseria meningitidis	W	11	ST-11 complex
31323	M1412	Chile		invasive (unspecified/other)	Neisseria meningitidis	W	11	ST-11 complex
34547	4119	Brazil	1989	invasive (unspecified/other)	Neisseria meningitidis	B	639	ST-32 complex
34548	9506	Brazil	2004	invasive (unspecified/other)	Neisseria meningitidis	C	33	ST-32 complex
34549	9757	Brazil	1988	invasive (unspecified/other)	Neisseria meningitidis	B	3597	ST-32 complex
34550	12888	Brazil	2004	invasive (unspecified/other)	Neisseria meningitidis	C	639	ST-32 complex
34570	73696	Brazil	1973	invasive (unspecified/other)	Neisseria meningitidis	discrepancy	8813	
34571	73704	Brazil	1973	invasive (unspecified/other)	Neisseria meningitidis	C	11	ST-11 complex
34572	75643	Brazil	1975	invasive (unspecified/other)	Neisseria meningitidis	A	5	ST-5 complex
34573	75689	Brazil	1975	invasive (unspecified/other)	Neisseria meningitidis	A	5	ST-5 complex
38176	M37087	Brazil	2014	carrier	Neisseria meningitidis	Z	11458	L2 complex
38177	M37113	Brazil	2014	carrier	Neisseria meningitidis	cnl	11459	ST-1136 complex
38178	M37959	Brazil	2014	carrier	Neisseria meningitidis	cnl	10238	ST-1136 complex

38179	M37128	Brazil	2014	carrier	Neisseria meningitidis	Y	11461	ST-23 complex
38180	M37958	Brazil	2014	carrier	Neisseria meningitidis	cnl	11462	ST-198 complex
39836	M03473	Argentina	1995		Neisseria meningitidis	C	11	ST-11 complex
39837	M05730	Brazil	1976		Neisseria meningitidis	C	11	ST-11 complex
39838	M13970	Brazil	2005		Neisseria meningitidis	C	8	ST-8 complex
39839	M13973	Brazil	2005		Neisseria meningitidis	C	5122	ST-103 complex
39840	M14431	Brazil	2005		Neisseria meningitidis	C	3779	ST-103 complex
54017	M37059	Brazil	2014		Neisseria meningitidis	E	10220	ST-254 complex, L1 complex
54018	M37060	Brazil	2014		Neisseria meningitidis	cnl	1136	ST-1136 complex
54019	M37064	Brazil	2014		Neisseria meningitidis	B	639	ST-32 complex
54020	M37065	Brazil	2014		Neisseria meningitidis	cnl	1136	ST-1136 complex
54021	M37067	Brazil	2014		Neisseria meningitidis	cnl	823	ST-198 complex
54022	M37068	Brazil	2014		Neisseria meningitidis	E	178	ST-178 complex
54023	M37069	Brazil	2014		Neisseria meningitidis	B	2120	ST-41/44 complex
54024	M37074	Brazil	2014		Neisseria meningitidis	cnl		
54025	M37082	Brazil	2014		Neisseria meningitidis	cnl	1136	ST-1136 complex
54026	M37087	Brazil	2014		Neisseria meningitidis	Z	11458	L2 complex
54027	M37089	Brazil	2014		Neisseria meningitidis	NG	6525	
54028	M37093	Brazil	2014		Neisseria meningitidis	cnl	1136	ST-1136 complex
54029	M37095	Brazil	2014		Neisseria meningitidis	cnl	11552	ST-1136 complex
54030	M37098	Brazil	2014		Neisseria meningitidis	E	10220	ST-254 complex, L1 complex
54031	M37099	Brazil	2014		Neisseria meningitidis	Y	23	ST-23 complex
54032	M37102	Brazil	2014		Neisseria meningitidis	cnl	823	ST-198 complex
54033	M37103	Brazil	2014		Neisseria meningitidis	Y	3015	
54034	M37104	Brazil	2014		Neisseria meningitidis	W	11	ST-11 complex
54035	M37108	Brazil	2014		Neisseria meningitidis	cnl	53	ST-53 complex
54036	M37111	Brazil	2014		Neisseria meningitidis	Z	2123	L2 complex
54037	M37112	Brazil	2014		Neisseria meningitidis	NG	639	ST-32 complex
54038	M37113	Brazil	2014		Neisseria meningitidis	cnl	11459	ST-1136 complex
54039	M37114	Brazil	2014		Neisseria meningitidis	cnl	823	ST-198 complex
54040	M37115	Brazil	2014		Neisseria meningitidis	B	11577	ST-41/44 complex
54041	M37116	Brazil	2014		Neisseria meningitidis	cnl	1136	ST-1136 complex
54042	M37118	Brazil	2014		Neisseria meningitidis	cnl	1136	ST-1136 complex
54043	M37121	Brazil	2014		Neisseria meningitidis	B	3200	ST-4821 complex
54044	M37122	Brazil	2014		Neisseria meningitidis	NG	6525	
54045	M37125	Brazil	2014		Neisseria meningitidis	cnl	7450	ST-1136 complex
54046	M37126	Brazil	2014		Neisseria meningitidis	cnl	7129	
54047	M37127	Brazil	2014		Neisseria meningitidis	E	10224	ST-254 complex, L1 complex
54048	M37128	Brazil	2014		Neisseria meningitidis	Y	11461	ST-23 complex
54049	M37133	Brazil	2014		Neisseria meningitidis	cnl	1136	ST-1136 complex

54050	M37134	Brazil	2014		Neisseria meningitidis	E	10220	ST-254 complex, L1 complex
54051	M37135	Brazil	2014		Neisseria meningitidis	cnl	13979	ST-198 complex
54052	M37139	Brazil	2014		Neisseria meningitidis	cnl	1136	ST-1136 complex
54053	M37141	Brazil	2014		Neisseria meningitidis	W	7097	ST-11 complex
54054	M37144	Brazil	2014		Neisseria meningitidis	B	3496	ST-213 complex
54055	M37148	Brazil	2014		Neisseria meningitidis	cnl	53	ST-53 complex
54056	M37150	Brazil	2014		Neisseria meningitidis	cnl	13327	ST-198 complex
54057	M37151	Brazil	2014		Neisseria meningitidis	cnl	53	ST-53 complex
54058	M37152	Brazil	2014		Neisseria meningitidis		5770	ST-175 complex
54059	M37153	Brazil	2014		Neisseria meningitidis	Z	5953	L2-complex
54060	M37154	Brazil	2014		Neisseria meningitidis	cnl	53	ST-53 complex
54061	M37958	Brazil	2014		Neisseria meningitidis	cnl	11462	ST-198 complex
54062	M37959	Brazil	2014		Neisseria meningitidis	cnl	10238	ST-1136 complex
54063	M37960	Brazil	2014		Neisseria meningitidis	cnl	53	ST-53 complex
56749	MCRJ 001	Brazil	2016	carrier	Neisseria meningitidis	B	13212	ST-41/44 complex
56750	MCRJ 074	Brazil	2016	carrier	Neisseria meningitidis	C	11	ST-11 complex
56751	MCRJ 107	Brazil	2016	carrier	Neisseria meningitidis	B	4221	
56752	MCRJ 108	Brazil	2016	carrier	Neisseria meningitidis	C	8730	
61385	M-011-16	Chile	2016	septicemia	Neisseria meningitidis	B	44	ST-41/44 complex
61386	M-015-16	Chile	2016	meningitis	Neisseria meningitidis	B	103	ST-103 complex
61387	M-023-16	Chile	2016	meningitis	Neisseria meningitidis	B	44	ST-41/44 complex
61388	M-046-16	Chile	2016	meningitis	Neisseria meningitidis	B	44	ST-41/44 complex
61389	M-047-16	Chile	2016	meningitis	Neisseria meningitidis	B	44	ST-41/44 complex
61556	M-009-16	Chile	2016	septicemia	Neisseria meningitidis	discrepancy	1025	ST-11 complex
61557	M-014-16	Chile	2016	meningitis	Neisseria meningitidis	W	11	ST-11 complex
61558	M-016-16	Chile	2016	septicemia	Neisseria meningitidis	W	11	ST-11 complex
61559	M-018-16	Chile	2016	septicemia	Neisseria meningitidis	W	11	ST-11 complex
61560	M-026-16	Chile	2016	septicemia	Neisseria meningitidis	discrepancy	2808	ST-103 complex
61561	M-064-16	Chile	2016	septicemia	Neisseria meningitidis	W	11	ST-11 complex
61562	M-067-16	Chile	2016	meningitis	Neisseria meningitidis	Y	11	ST-11 complex
61563	M-079-16	Chile	2016	septicemia	Neisseria meningitidis	W	11	ST-11 complex
61564	M-092-16	Chile	2016	septicemia	Neisseria meningitidis	W	11	ST-11 complex
61565	M-103-16	Chile	2016	septicemia	Neisseria meningitidis	B	11	ST-11 complex
64032	M42473	Brazil	2016	carrier	Neisseria meningitidis	E	6119	
93479	M42412	Brazil	2016	carrier	Neisseria meningitidis	cnl	1136	ST-1136 complex
93480	M42413	Brazil	2016	carrier	Neisseria meningitidis	B	437	ST-41/44 complex
93481	M42414	Brazil	2016	carrier	Neisseria meningitidis	cnl	7129	
93482	M42415	Brazil	2016	carrier	Neisseria meningitidis	cnl	3876	ST-198 complex
93483	M42416	Brazil	2016	carrier	Neisseria meningitidis	cnl	11312	
93484	M42418	Brazil	2016	carrier	Neisseria meningitidis	Z	13102	L2-complex
93485	M42419	Brazil	2016	carrier	Neisseria meningitidis	cnl	1136	ST-1136 complex
93486	M42420	Brazil	2016	carrier	Neisseria meningitidis	cnl	1136	ST-1136 complex

93487	M42421	Brazil	2016	carrier	Neisseria meningitidis	cnl	823	ST-198 complex
93488	M42424	Brazil	2016	carrier	Neisseria meningitidis	Z	13103	L2 complex
93489	M42425	Brazil	2016	carrier	Neisseria meningitidis	cnl	7129	
93490	M42426	Brazil	2016	carrier	Neisseria meningitidis	B	437	ST-41/44 complex
93491	M42427	Brazil	2016	carrier	Neisseria meningitidis	cnl	1136	ST-1136 complex
93492	M42428	Brazil	2016	carrier	Neisseria meningitidis	cnl	7129	
93493	M42429	Brazil	2016	carrier	Neisseria meningitidis	B	269	ST-269 complex
93494	M42431	Brazil	2016	carrier	Neisseria meningitidis	cnl	7129	
93495	M42435	Brazil	2016	carrier	Neisseria meningitidis	cnl	823	ST-198 complex
93496	M42441	Brazil	2016	carrier	Neisseria meningitidis	Z	5953	L2 complex
93497	M42442	Brazil	2016	carrier	Neisseria meningitidis	W	13104	
93498	M42444	Brazil	2016	carrier	Neisseria meningitidis	cnl	13105	ST-1136 complex
93499	M42445	Brazil	2016	carrier	Neisseria meningitidis	B	2160	ST-750 complex, L2 complex
93500	M42446	Brazil	2016	carrier	Neisseria meningitidis	cnl	7129	
93501	M42448	Brazil	2016	carrier	Neisseria meningitidis	cnl	13106	ST-1136 complex
93502	M42450	Brazil	2016	carrier	Neisseria meningitidis	cnl	823	ST-198 complex
93503	M42451	Brazil	2016	carrier	Neisseria meningitidis	cnl	7129	
93504	M42454	Brazil	2016	carrier	Neisseria meningitidis	cnl	13107	
93505	M42456	Brazil	2016	carrier	Neisseria meningitidis	cnl	823	ST-198 complex
93506	M42458	Brazil	2016	carrier	Neisseria meningitidis	B	3200	ST-4821 complex
93507	M42459	Brazil	2016	carrier	Neisseria meningitidis	cnl	823	ST-198 complex
93508	M42481	Brazil	2016	carrier	Neisseria meningitidis	cnl	10238	ST-1136 complex
93509	M42460	Brazil	2016	carrier	Neisseria meningitidis	E	10220	ST-254 complex, L1 complex
93510	M42461	Brazil	2016	carrier	Neisseria meningitidis	B	3327	ST-865 complex
93511	M42467	Brazil	2016	carrier	Neisseria meningitidis	cnl	3876	ST-198 complex
93512	M42468	Brazil	2016	carrier	Neisseria meningitidis	cnl	823	ST-198 complex
93513	M42470	Brazil	2016	carrier	Neisseria meningitidis	B	409	ST-41/44 complex
93514	M42471	Brazil	2016	carrier	Neisseria meningitidis	B	1572	ST-1572 complex
93515	M42475	Brazil	2016	carrier	Neisseria meningitidis	cnl	1136	ST-1136 complex
93516	M42476	Brazil	2016	carrier	Neisseria meningitidis	B	409	ST-41/44 complex
93517	M42477	Brazil	2016	carrier	Neisseria meningitidis	E	1157	ST-1157 complex
93518	M42478	Brazil	2016	carrier	Neisseria meningitidis	cnl	823	ST-198 complex
93519	M42479	Brazil	2016	carrier	Neisseria meningitidis	cnl	823	ST-198 complex
93520	M42480	Brazil	2016	carrier	Neisseria meningitidis	B	11827	ST-35 complex
93521	M42483	Brazil	2016	carrier	Neisseria meningitidis	cnl	823	ST-198 complex
93522	M42484	Brazil	2016	carrier	Neisseria meningitidis	cnl	823	ST-198 complex
93523	M42485	Brazil	2016	carrier	Neisseria meningitidis	cnl	53	ST-53 complex
93524	M42469	Brazil	2016	carrier	Neisseria meningitidis	cnl	7129	

Table S2. Details of predicted prophages

Proposed Name	Species *	Genus*	Family*	Size	Nº of genes	Type of virus	Genome Quality	Taxonomy **	Reference genome (PubMLST id)
NmSA1	6	G3	F3	6,85 Kb	6	Lytic	Low		26035
NmSA2	11	G4	F1	8,98 Kb	18	Lytic	Medium		31322
NmSA3	7	G4	F1	7,18 Kb	11	Lytic	Circular		31322
NmSA4	40	G8	F3	37,4 Kb	55	Lytic	High	Myoviridae	34549
NmSA5	48	G12	F3	41,4 Kb	55	Lysogenic	Medium		34570
NmSA6	31	G8	F3	6,85 Kb	6	Lytic	Low	Myoviridae	34572
NmSA7	1	G8	F3	8,98 Kb	18	Lytic	Medium		34572
NmSA8	12	G8	F3	9,43 Kb	0	-	-		38176
NmSA9	42	G8	F3	39,0 Kb	58	Lytic	High	Myoviridae	38177
NmSA10	37	G8	F3	32,9 Kb	49	Lytic	Medium	Myoviridae	38179
NmSA11	19	G11	F3	11,5 Kb	13	Lytic	Low		38180
NmSA12	18	G6	F3	4,05 Kb	10	Lytic	Low		38180
NmSA13	15	G7	F3	9,92 Kb	15	Lysogenic	Low		39838
NmSA14	52	G11	F3	43,3 Kb	61	Lysogenic	High		39840
NmSA15	41	G10	F3	38,0 Kb	53	Lysogenic	Medium		39840
NmSA16	21	G14	F3	5,0 Kb	13	Lytic	Medium		54028
NmSA17	10	G5	F3	3,28Kb	10	Lytic	Low		54028
NmSA18	56	G14	F3	5,21 Kb	13	Lytic	Low		54028
NmSA19	34	G8	F3	25,8 Kb	30	Lytic	Medium	Myoviridae	54028
NmSA20	16	G7	F3	10,1 Kb	17	Lysogenic	Low		54033
NmSA21	17	G7	F3	10,3 Kb	16	Lysogenic	Medium		54034
NmSA22	45	G10	F3	40,9 Kb	53	Lysogenic	Medium		54034
NmSA23	46	G12	F3	41,2 Kb	54	Lysogenic	Medium		54036
NmSA24	50	G9	F3	2,18 Kb	5	Lytic	Low		54043
NmSA25	43	G14	F3	4,86 Kb	12	Lytic	Low		54043
NmSA26	26	G8	F3	19,8 Kb	26	Lytic	Medium	Myoviridae	54054
NmSA27	39	G11	F3	36,2 Kb	55	Lysogenic	Medium		54056
NmSA28	28	G8	F3	22,8 Kb	31	Lytic	Medium	Myoviridae	54058
NmSA29	25	G11	F3	19,7 Kb	33	Lytic	Medium		54059
NmSA30	22	G11	F3	15,2 Kb	21	Lysogenic	Medium		54059
NmSA31	24	G8	F3	19,3 Kb	31	Lytic	Medium	Myoviridae	56749

NmSA32	55	G13	F2	1,21 Kb	4	Lytic	Low		61558
NmSA33	4	G7	F3	6,43 Kb	12	Lysogenic	Low		61560
NmSA34	32	G13	F2	2,64 Kb	5	Lytic	Low		64032
NmSA35	30	G11	F3	24,6 Kb	37	Lytic	Low		650
NmSA36	3	G2	F2	5,39 Kb	8	Lytic	Circular		93479
NmSA37	14	G5	F3	9,83 Kb	20	Lytic	Low		93483
NmSA38	5	G8	F3	6,57 Kb	9	Lytic	Low		93484
NmSA39	33	G14	F3	3,5 Kb	8	Lytic	Low		93486
NmSA40	38	G11	F3	35,3 Kb	50	Lytic	Medium		93488
NmSA41	13	G7	F3	9,53 Kb	18	Lysogenic	Low		93493
NmSA42	2	G1	F3	5,38 Kb	5	Lytic	Low		93496
NmSA43	8	G7	F3	7,85 Kb	14	Lysogenic	Low		93496
NmSA44	51	G10	F3	39,2 Kb	48	Lysogenic	High		93498
NmSA45	49	G12	F3	41,7 Kb	54	Lysogenic	Medium		93499
NmSA46	9	G7	F3	7,89 Kb	15	Lysogenic	Low		93506
NmSA47	23	G8	F3	15,9 Kb	24	Lytic	Low	Myoviridae	93506
NmSA48	47	G10	F3	41,3 Kb	56	Lysogenic	High		93508
NmSA49	36	G11	F3	32,0 Kb	49	Lytic	Medium		93511
NmSA50	20	G7	F3	11,6 Kb	19	Lysogenic	Baja		93513
NmSA51	27	G8	F3	21,7 Kb	31	Lytic	High	Myoviridae	93513
NmSA52	44	G12	F3	40,4 Kb	50	Lysogenic	Medium		93514
NmSA53	35	G11	F3	31,7 Kb	51	Lytic	Medium		93515
NmSA54	53	G12	F3	58,1 Kb	65	Lysogenic	Medium		93517
NmSA55	54	G14	F3	1,33 Kb	4	Lytic	Medium		93522
NmSA56	29	G8	F3	23,9 Kb	32	Lytic	Medium		93523

* Determined by VICTOR (https://ggdc.dsmz.de/victor.php)
** Inferred by vConTACT2 (https://bitbucket.org/MAVERICLab/vcontact2/wiki/Home)

Table S3. Gene annotation of IMSAR-11 prophage based on 38VI genome

Predicted Protein	Size (aa)	Predicted Function	E Value	Reference
Repressor protein C	109	Allows the phage to reside inactively in the chromosome of its host bacterium.	4.3E-4	Uniprot ID: P69202
Hypothetical	105	-	22*	-
Hypothetical	62	-	1.9*	-

Hypothetical	79	-	4.9*	-
rstA1	390	Necessary for the initiation of phage replication.	4.3E-30	PDB ID: 4CIJ
Hypothetical	94	-	27*	-
G5P	100	DNA Binding.<br>The capsid protein G8P displaces it during phage assembly on the inner bacterial membrane.	3.2E-3	Uniprot ID: P03671
G8P	75	Self assembles to form a helical capsid wrapping up the viral genomic DNA.	4.2E-2	Uniprot ID: P03621
G8P	93	Self assembles to form a helical capsid wrapping up the viral genomic DNA.	3.5E-11	Uniprot ID: P03621
Hypothetical	94	Hypothetical	8.7*	--
G3P	329	Viral attachment to the host cell mediates the adsorption of the phage to its primary receptor.<br>Has a role in viral extrusion.	1.1e-8	Uniprot ID: P03624
G6P	96	It plays essential roles both in entering the viral genome into the bacterial host and in the budding process.	2.6E-19	Uniprot ID: P03625
G1P	361	It may play a role in phage assembly.<br>ZOT homolog.	5E-23	Uniprot ID: P03626
* These values were not sufficient to assign a probable function				

Table S4. BLAST results of the worldwide presence of IMSAR-11 (NmSA3 from M20599)

(A). BLAST results of the presence of IMSAR-11 in genomes sequenced by continent

**Continent**	**Presence in total genomes**	**Presence in carrier genomes**	**Presence in invasive genomes**	**% of invasiveness**	**Presence in the total of invasive genomes**	**% of presence in the total of invasive genomes**	**Query coverage limit**	**Identity limit**
South America	179/442	1/179	178/179	99.44	178/255	69.80	92,00%	98,79%
North America	847/3190	15/847	429/847	50.65	429/1913	22,43	90,37%	98,56%
Asia	43/1037	4/43	18/43	41.86	18/492	3.66	95,54%	99,12%
Europe	2696/17778	216/2696	2079/2696	77.11	2079/10473	19.85	90,00%	98,38%
Africa	193/3959	4/193	158/193	81.87	158/1848	8.55	93,55%	99,06
Oceania	152/710	0/152	142/152	93.42	142/603	23.55	92,89%	99,12%

(B). Distribution of IMSAR-11 by clonal complex (cc)							
**Continent**	**cc8 N° genomes**	**cc11 N° genomes**	**cc22 N° genomes**	**cc23 N° genomes**	**cc32 N° genomes**	**cc41/44 N° genomes**	**cc1157 N° genomes**
South America	1	175	0	0	0	0	0
North America	0	843	0	0	1	1	1
Asia	1	42	0	0	0	0	0
Europe	38	2603	1	1	1	1	0
Africa	0	187	0	0	0	0	0
Oceania	0	152	0	0	0	0	0
Total	**40**	**4002**	**1**	**1**	**2**	**2**	1
%	0.99	98.84	0.02	0.02	0.05	0.05	0.02

(C). Distribution of IMSAR-11 by serogroup				
**Continent**	**Serogroup B**	**Serogroup C**	**Serogroup W**	**Serogroup Y**
South America	1	31	144	1
North America	16	532	288	6
Asia	1	26	14	1
Europe	101	734	1800	6
Africa	0	29	156	0
Oceania	0	39	111	0
Total	**119**	**1391**	**2513**	**14**
%	2.95	34.46	62.25	0.35

(D). Prevalence of IMSAR-11 in the cc11				
**Continent**	**IMSAR-11 presence in cc11 (N° genomes)**	**% Of presence in cc11 genomes**	**Query cover % limit**	**% Identity limit**
South America	175/198	88,38	91,69	98,79
North America	843/971	86,82	95,54	98,56
Asia	42/99	42,42	95,54	99,12
Europe	2603/3962	65,70	90,00	98,47
Africa	190/872	21,79	94,55	99,06

Oceania	152/174	87,36	92,89	99,12

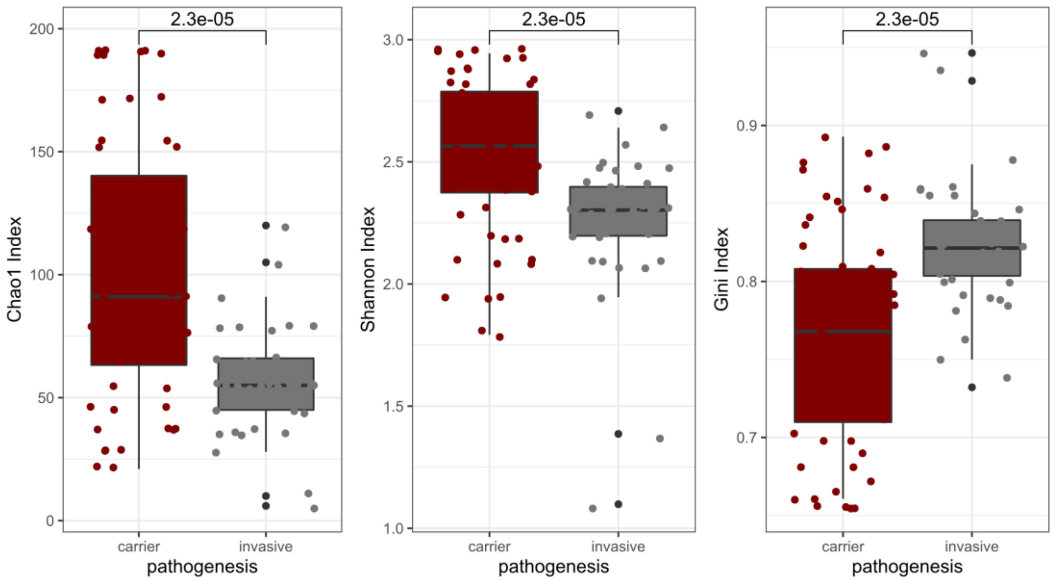

Figure S1. Alpha diversity of phages according to the pathogenicity of *N. meningitidis* strains from South America.

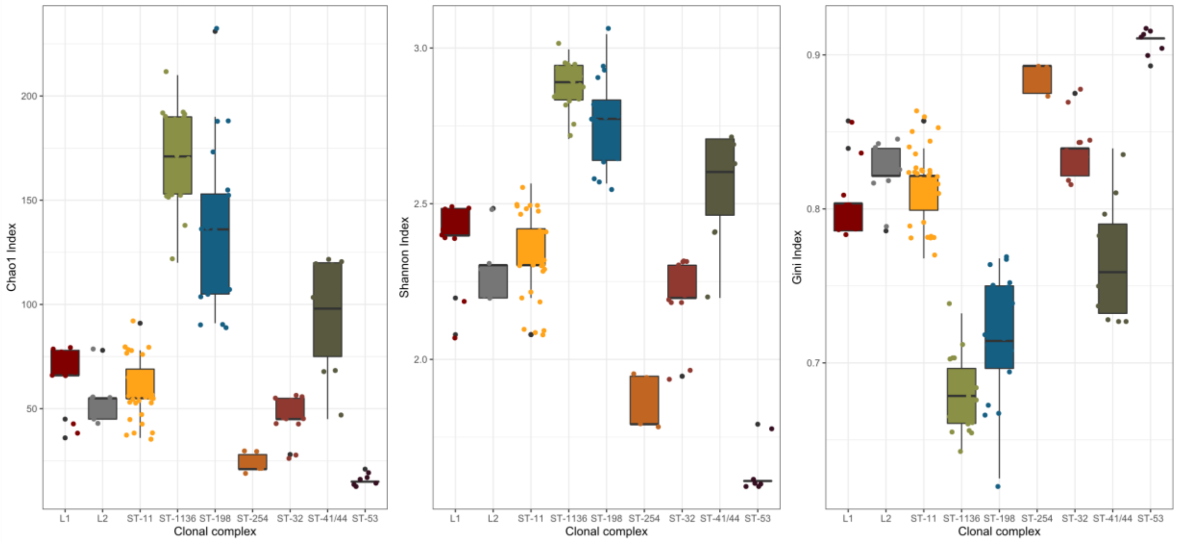

Figure S2. Alpha diversity of phages according to clonal-complex of *N. meningitidis* strains.

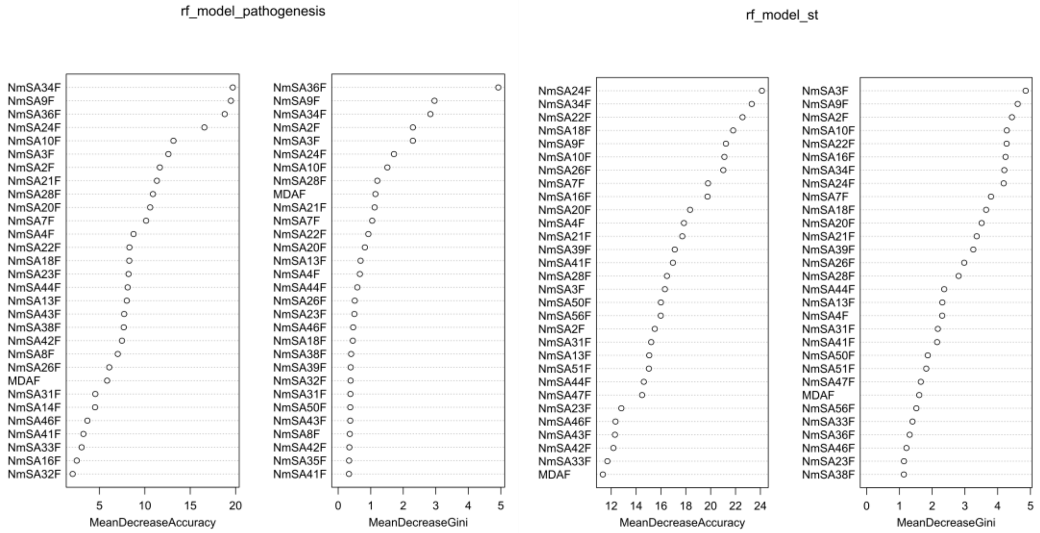

Figure S3. Random-Forest's Predictors. On the left top predictor phages of pathogenicity. On the right, top predictor phages for the clonal complex.

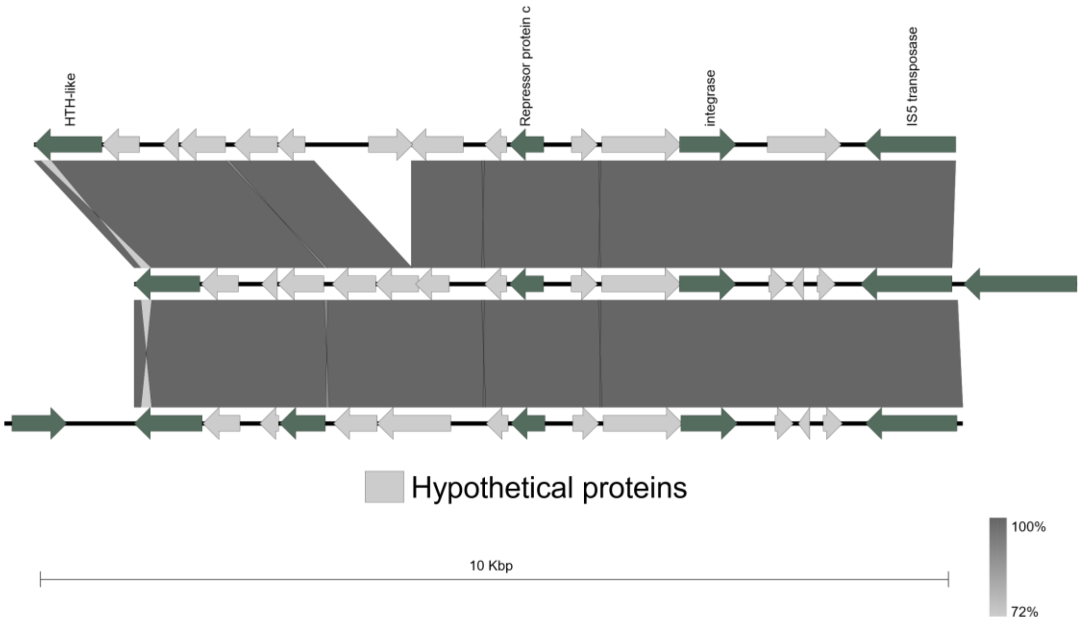

Figure S4. DNA Comparison between NmSA13 (at the top), NmSA20 (at the middle), and NmSA21 (at the bottom). These phages were widely distributed in the genomes of *N. meningitidis*.

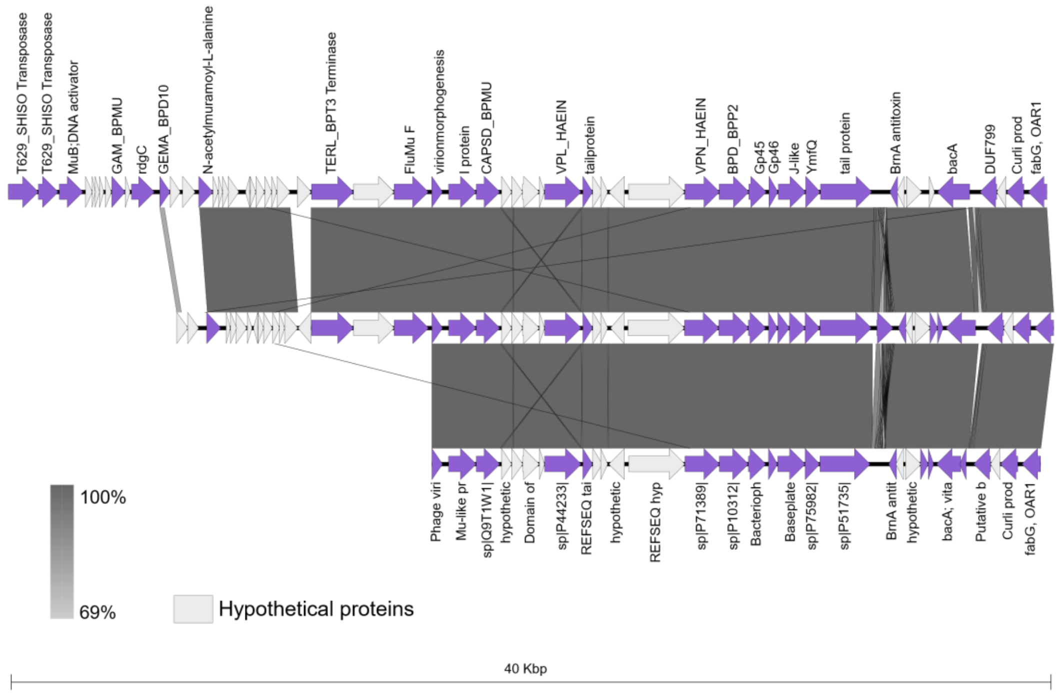

Figure S5. Comparison between NmSA9 (At the top), NmSA10 (at the middle), and NmSA28 (at the bottom). These phages were widely distributed in the commensal genomes of *N. meningitidis.* The comparison was carried out considering DNA sequences.

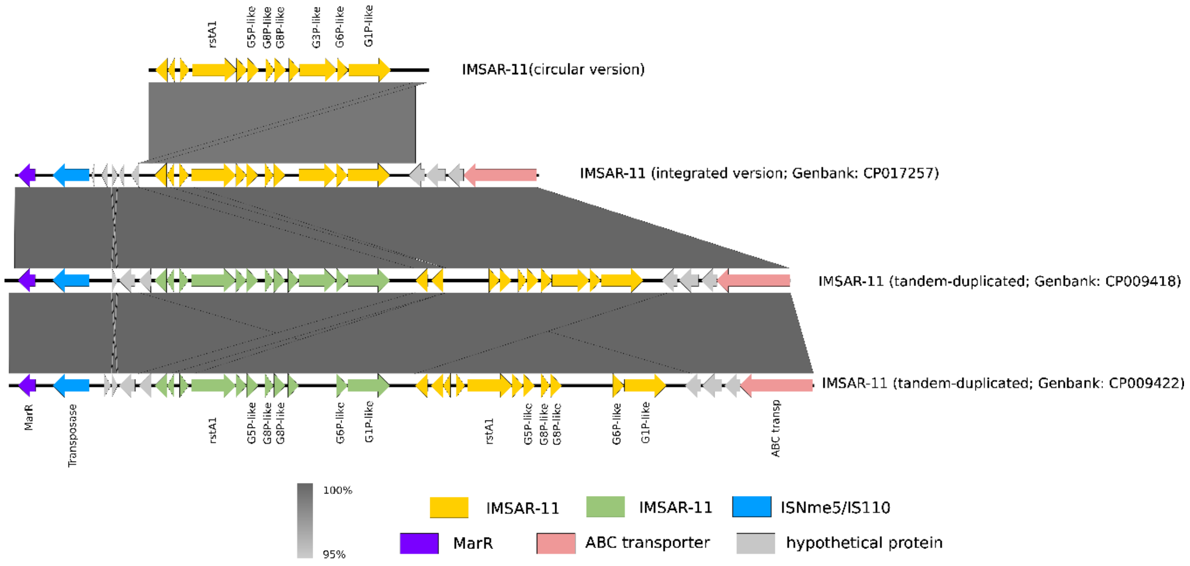

Figure S6. Genomic context of IMSAR-11. Comparison of the genetic structure of IMSAR-11 found in other genomes with NmSA3 (circular version of IMSAR-11 in strain M20599).

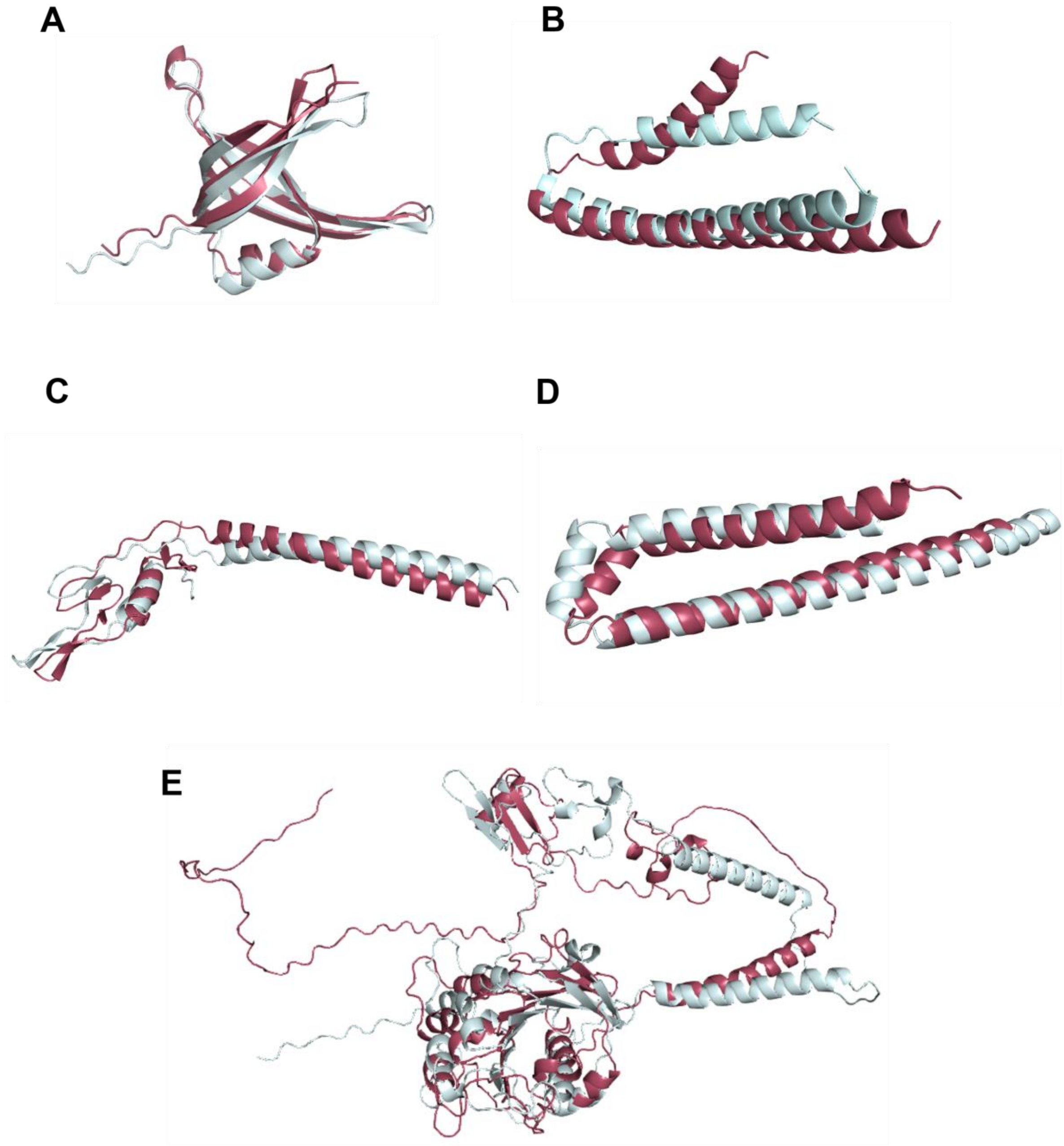

Figure S7. Comparison of protein structures of MDA/Nf1 (strain: Z2491) (depicted in pale red) and protein structures of IMSA-11 (strain: 38VI) (shown in pale cyan). (**A**) The alignment between ORF2 and G5P-like obtained an RMSD of 0.869 Å. (**B**) The alignment between ORF4 and G8Pa-like obtained an RMSD of 4.990 Å. (**C**) The alignment between ORF5 and G8Pb-like obtained an RMSD of 3.762 Å. (**D**) The alignment between ORF7 and G6P-like obtained an RMSD of 4.737 Å. (**E**) The alignment between ORF8 and G1P-like obtained an RMSD of 6.241 Å. The rest of the proteins showed low confidence or similarity in the structural prediction.

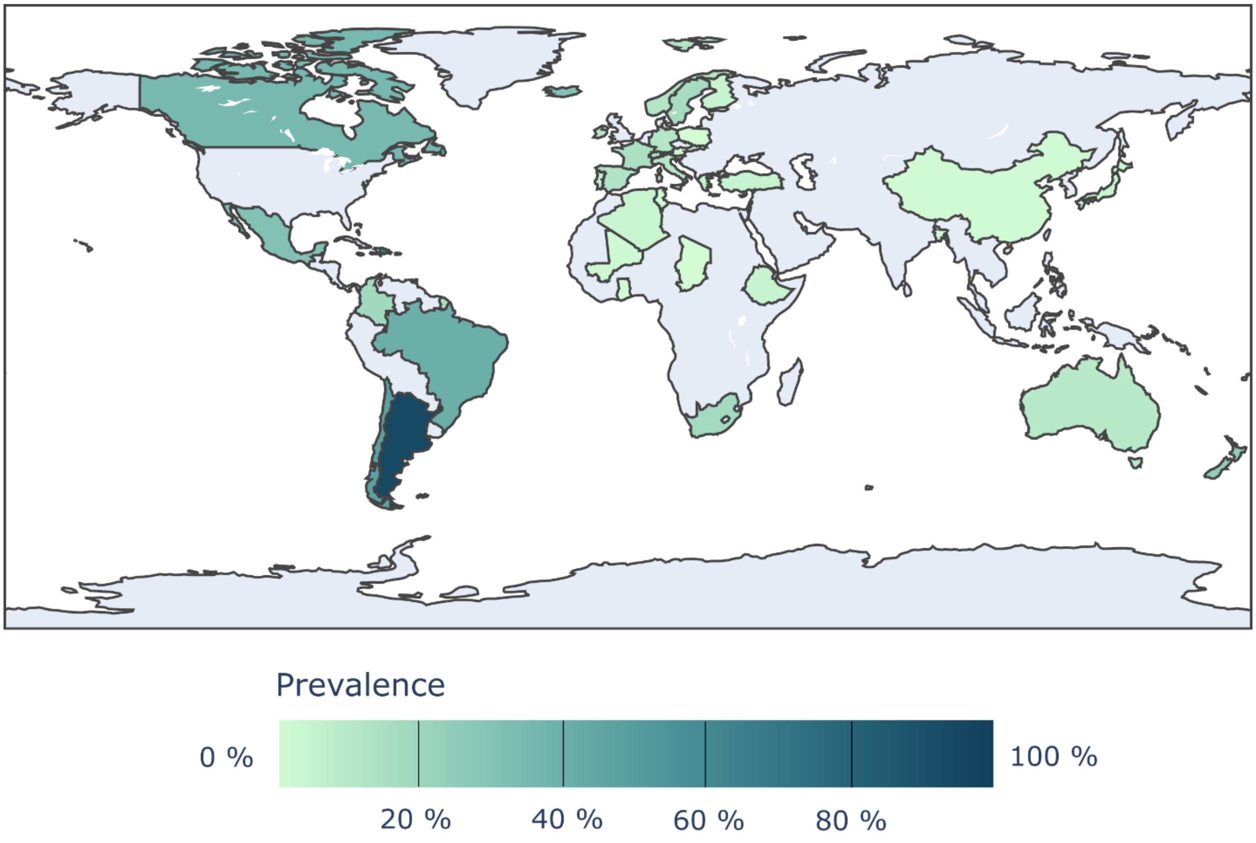

Figure S8. The global prevalence of IMSAR-11. The prevalence of IMSAR-11 is the relationship between the presence of IMSAR-11 and the total number of genomes sequenced by country.
